# Interstitial Lung Disease in Patients With Systemic Sclerosis: Toward Personalized-Medicine-Based Prediction and Drug Screening Models of Systemic Sclerosis-Related Interstitial Lung Disease (SSc-ILD)

**DOI:** 10.3389/fimmu.2020.01990

**Published:** 2020-09-04

**Authors:** Padmini Khedoe, Emiel Marges, Pieter Hiemstra, Maarten Ninaber, Miranda Geelhoed

**Affiliations:** Department of Pulmonology, Leiden University Medical Center (LUMC), Leiden, Netherlands

**Keywords:** systemic sclerosis, interstitial lung disease, idiopathic pulmonary fibrosis, pathogenesis, organoids, human disease models

## Abstract

Systemic sclerosis (SSc) is an autoimmune connective tissue disease, characterized by immune dysregulation and progressive fibrosis. Interstitial lung disease (ILD) is the most common cause of death among SSc patients and there are currently very limited approved disease-modifying treatment options for systemic sclerosis-related interstitial lung disease (SSc-ILD). The mechanisms underlying pulmonary fibrosis in SSc-ILD are not completely unraveled, and knowledge on fibrotic processes has been acquired mostly from studies in idiopathic pulmonary fibrosis (IPF). The incomplete knowledge of SSc-ILD pathogenesis partly explains the limited options for disease-modifying therapy for SSc-ILD. Fibrosis in IPF appears to be related to aberrant repair following injury, but whether this also holds for SSc-ILD is less evident. Furthermore, immune dysregulation appears to contribute to pro-fibrotic responses in SSc-ILD, perhaps more than in IPF. In addition, SSc-ILD patient heterogeneity complicates the understanding of the underlying mechanisms of disease development, and more importantly, limits correct clinical diagnosis and treatment effectivity. Therefore, there is an unmet need for patient-relevant (*in vitro*) models to examine patient-specific disease pathogenesis, predict disease progression, screen appropriate treatment regimens and identify new targets for treatment. Technological advances in *in vitro* patient-relevant disease modeling, including (human induced pluripotent stem cell (hiPSC)-derived) lung epithelial cells, organoids and organ-on-chip technology offer a platform that has the potential to contribute to unravel the underlying mechanisms of SSc-ILD development. Combining these models with state-of-the-art analysis platforms, including (single cell) RNA sequencing and (imaging) mass cytometry, may help to delineate pathogenic mechanisms and define new treatment targets of SSc-ILD.

## Introduction

Systemic sclerosis (SSc) is a devastating disease of unknown etiology, characterized by systemic, immunological, vascular, and fibrotic abnormalities, with a heterogeneous clinical course. Fibrosis, the hallmark of the disease, can affect skin and internal organs, including the lung (1). SSc-related interstitial lung disease (SSc-ILD) is one of the most severe complications and is the main cause of SSc-related deaths (2, 3). Treatment options are limited, although the FDA has recently approved nintedanib for progressive SSc-ILD. The limitation in options for disease-modifying treatments may partly be explained by the incomplete knowledge of the mechanisms underlying lung fibrosis development in SSc-ILD and the heterogeneous course of disease. Furthermore, the optimal type and timing of treatment of SSc-ILD is unknown and it is becoming increasingly clear that the treatment of SSc-ILD patients requires a personalized approach. An unknown subgroup of patients with SSc may benefit from antifibrotic drugs such as nintedanib and related compounds, in addition to their immunosuppressive therapy. Therefore, there is an unmet need for novel biomarkers to predict disease progression and response to treatment (4). As a result, the prognosis for SSc-ILD patients remains extremely poor, and there is an urgent need for better disease-relevant models to identify new treatment targets and for (personalized) drug screening.

Although various experimental animal models of human ILD are currently available, they do not fully represent human disease development, and fibrosis is often reversible (5). Furthermore, whereas pathogenic mechanisms can be studied to some extent using inflammatory cells derived from peripheral blood and bronchoalveolar lavage (BAL) from SSc-ILD patients, insight into the interaction between key players in the pathogenesis of fibrosis is often lacking. Currently, there are only a few available preclinical human *in vitro* models which recapitulate features of fibrosis, including the complex interplay between (myo)fibroblasts, epithelial cells, inflammatory cells and endothelial cells, and these are reviewed elsewhere (5). The extent to which these models recapitulate fibrosis development in SSc-ILD is unclear.

**FIGURE 1:**
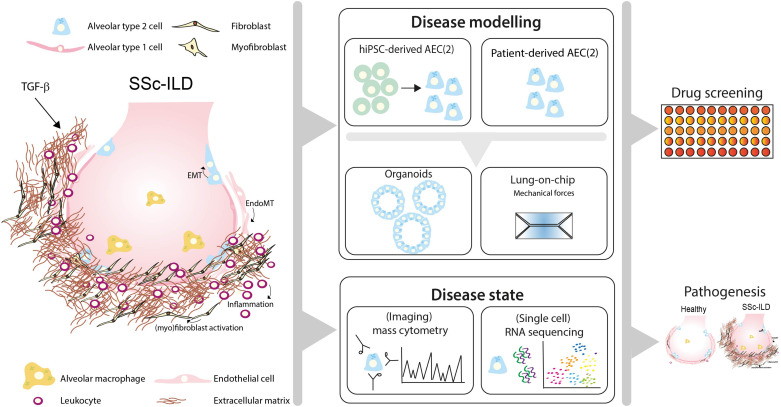
Toward personalized-medicine-based prediction, target identification and drug screening models of SSc-ILD. In SSc-ILD, the interactions and cross-talk between various cell types, including in type 2 alveolar cells (AEC2), AEC1, endothelial cells, fibroblasts and immune cells is disturbed. This may be partly explained by excessive TGF-β signaling activation, leading to AEC1/2 and endothelial cell (EC) transition into a mesenchymal phenotype, (myo)fibroblast activation and proliferation and excessive interstitial extracellular matrix (ECM) deposition. Recruited leukocytes further contribute to the profibrotic environment, leading to lung tissue remodeling and fibrosis. Patient-derived pulmonary cells, either isolated from lung tissue or derived from human induced pluripotent stem cells (hiPSC), can be used to study patient-specific responses *in vitro* using organoid SSc-ILD disease models. Lung-chip models may add another layer of complexity containing physiological ques, including breathing motions, air-blood flow and stretch, which are often lacking in current available *in vitro* lung models. Disease-specific features can be measured using cutting-edge techniques such as single-cell-RNA-sequencing and (imaging) mass cytometry. Integrating information derived from SSc-ILD disease models using cells derived from SSc-ILD patients and triggers implicated in disease pathogenesis may lead to novel mechanistic insight and offer a platform for identification of pathogenic processes and drug screening for SSc-ILD.

Here, we discuss how recent advances in human preclinical models may help to unravel pathogenic mechanisms and new treatment targets of SSc-ILD by shortly reviewing the cellular processes that lead to fibrosis in IPF and SSc-ILD (Figure 1).

## Common Fibrotic Pathways

SSc-ILD can be regarded as an impaired or otherwise aberrant repair response in the alveolar areas. Proposed mechanisms of fibrosis development and progression include profibrotic responses following lung alveolar epithelial cell (AEC) damage, aberrant alveolar repair (6, 7) and accelerated lung epithelial aging (8). Impaired regenerative capacity of lung progenitor cells, mainly the type 2 alveolar cells (AEC2), are suggested to be key players in the aberrant repair response. In concert with activated endothelial cells (EC) and recruited inflammatory cells, AEC2 orchestrate a profibrotic niche leading to fibroblast activation, myofibroblast formation and excessive extracellular matrix (ECM) deposition. Increased numbers of (myo)fibroblasts and their activation is mainly driven by transforming growth factor-β (TGF-β), whereas in alveolar epithelial type 1 and 2 cells (AEC1/2) and EC, excessive TGF-β may result in epithelial-to-mesenchymal transition (EMT) and endothelial-to-mesenchymal transition (endoMT) (9–12). TGF-β, produced by various cells types, is the central regulator of fibrotic processes and its levels are increased in both IPF and SSc-ILD lungs (13, 14). TGF-β enhances expression of profibrotic and pro-inflammatory cytokines and growth factors, such as tumor necrosis factor-α (TNF-α), platelet derived growth factor (PDGF), Interleukin-1β (IL-1β), and IL-13 (15). Target genes of TGF-β include e.g., α-smooth muscle actin (α-SMA) and connective tissue growth factor (CTGF), which are increased in fibrosis (12). Plasma CTGF levels were reported to be elevated in IPF patients (16) and, together with Vascular Endothelial Growth Factor (VEGF) and TGF-β, act as central mediators in fibrosis (14, 17–19). Altogether, the profibrotic niche leads to aberrant alveolar repair responses, fibroblast foci and excessive extracellular matrix (ECM) production, causing extensive pulmonary remodeling resulting in impairment of lung function.

Since one of the key players in this aberrant repair response is the AEC2, lung organoid assays provide a tool to study the regenerative ability of these lung progenitor cells. Organoids are three-dimensional (3D) self-organizing structures of multiple cell types grown in 3-D matrices, as opposed to a 2D cell culture on culture plates. The 3D matrix contains a mixture of ECM proteins and various growth factors, necessary for expansion, proliferation and differentiation of lung epithelial (progenitor) cells (20). Lung organoids can be cultured for prolonged periods of time, currently up to 2 years, without changes in their karyotype, whilst preserving original (patient) characteristics. An important feature of organoids is that progenitor cells are retained in culture, and have the capacity to proliferate and differentiate into various specialized cell types (21). Furthermore, when generating lung epithelial organoids, the differentiation into bronchial, alveolar or mixed cell types can be achieved by selection of cells, manipulating the cultures with cocktails of cytokines and growth factors, stimulating or inhibiting signaling pathways, and/or by including mesenchymal cells that provide supporting signals for differentiation. Organoid differentiation and behavior may alter in response to a variety of injuries including exposure to SSc-ILD-related cytokines, infectious agents, tobacco smoke, high levels of oxygen and bleomycin (22, 23). This way, lung organoid models are well suited to study early phases of the fibrotic process. TGF-β treatment of airway organoids, for example, resulted in enhanced αSMA expression, and upregulated pro-fibrotic genes including CTGF and fibronectin (24, 25). Importantly, the complexity of the cross-talk between epithelial cells, fibroblasts and inflammatory cells that occur in ILD, can be mimicked in organoid models containing epithelial cells and surrounding cells, including fibroblasts and endothelial cells. This is also illustrated by a study showing that pretreatment of MRC-5 fibroblasts with TGF-β decreases their ability to support mouse epithelial organoid formation (26). Finally, recent advances allowing incorporation of inflammatory cells in lung organoid cultures may contribute to patient-relevant SSc-ILD models.

## Inflammation and Immunity

Fibrosis in SSc-ILD may result from an interplay between autoimmunity, inflammation, alveolar epithelial- and vascular injury (27), which may result in alveolitis. The alveolar and microvascular injurious events drive activation and proliferation of lung-resident immune cells and recruitment of inflammatory cells, including monocytes, neutrophils, mast cells and NK cells (28, 29). Interestingly, monocytes derived from SSc-ILD patients, which were increased in both the circulation and in lungs, exhibit pro-fibrotic properties *in vitro* upon pro-inflammatory stimulation (30). In addition, fibrotic mediators in SSc-ILD, including fibronectin-EDA and tenascin-C, may trigger activation of Toll Like Receptor 4 (TLR4) (31, 32) and cause uncontrolled ECM deposition in SSc (33), which subsequently potentiates TGF-β signaling, thereby enhancing fibrotic responses (15, 34).

Circulating and pulmonary (auto-reactive) B lymphocytes are important features in SSc-ILD (28, 35, 36) and produce pro-fibrotic mediators including TGF-β and Th2 cytokines (28). Furthermore, auto-antibodies, which are a characteristic feature of SSc, are produced by these autoreactive B cells and may cause local pulmonary damage and are associated with distinct clinical phenotypes of SSc (37). Development of ILD in SSc patients is strongly correlated with the presence of auto-antibodies against topoisomerase I, U11/12, Th/To,PL-7, U1-RNP, whereas anti-RNA polymerase III antibodies are associated with reduced risk of ILD in SSc (38). Also, anti-C1q antibodies were shown in a subgroup of SSc-ILD patients, and were found to be the most important predicting factor for pulmonary fibrosis development in SSc (39). Collectively, especially in SSc-ILD patients these autoreactive B cells may contribute to a profibrotic environment.

In IPF, the contribution of inflammation to IPF pathogenesis is less clear. Whereas the proportion of T- and B- cells was similar in IPF lungs compared to healthy lungs, both CD71^–^ alveolar- and S*PP1*/osteopontin^+^ interstitial macrophages were found to be associated with disease severity (40, 41), suggesting that lung-resident macrophages contribute to fibrosis in IPF. In addition, circulating monocytes in patients with IPF appear to be programmed to express fibroblast-stimulating properties prior to entering the lung (42). The contribution of macrophage subtypes in fibrosis development is controversial. Whereas pro-inflammatory M1 macrophages may contribute to dysregulated repair (e.g., in acute exacerbations of IPF) (43, 44), anti-inflammatory M2 macrophages may promote fibroproliferation and progressive fibrosis (42, 44). Furthermore, dysregulated apoptotic cell clearance and imbalanced ECM production and degradation by macrophages may contribute to fibrosis (45, 46).

In addition to enhanced levels of pro-fibrotic mediators, levels of pro-inflammatory cytokines and chemokines (including IL-8, IL-1α, IL-10, macrophage inflammatory protein-1α and MCP-1) and neutrophil-derived alpha-defensins (human neutrophil peptides (HNP), are increased in BALF from SSc-ILD patients (29, 47). In addition, also circulating levels of pro- and anti-inflammatory mediators, including IL-6, IL-10, MCP-1, IL-1RA and TNFα, were found to be increased. Some of these mediators correlate significantly with disease-relevant parameters (47, 48), and especially serum IL-6 is increased in both SSc-ILD and IPF, and may serve as a predictor of fibrosis progression (49). Recent studies intriguingly suggested both in IPF and SSc-ILD patients, an inverse correlation between lung function and myeloid-derived suppressor cell (MDSC) levels, highlighting their potential contribution to lung fibrosis (50). Thus, targeting the MDSC activity may be a novel approach to disease modification, and combined organoid models of epithelial and inflammatory cells may be useful tools to study this. This is illustrated by a study showing that lung organoids, comprised of epithelial and mesenchymal cells, respond to TLR- stimulation by releasing pro−inflammatory cytokines. Addition of primary human monocytes to these stimulated organoids, resulted in their migration toward the organoids and altered their phenotype to an “intermediate−like” phenotype, expressing increased levels of CD14, CD16 and HLA-DR compared to monocytes cultured alone (51).

Collectively these studies show that there are differences in the proposed role of inflammatory and immune cells in the pathogenesis of SSc-ILD and IPF. More complex organoid models, as outlined above, may help to further dissect the specific role of these cells, and the contribution of profibrotic and pro-inflammatory triggers in SSC-ILD. Indeed, whereas incorporation of patient-derived inflammatory and immune cells in organoids is logistically challenging, this may potentially provide preclinical personalized prediction models of disease development, and models for drug screening. In the tumor field, co-culture models of immune cells and tumor cells are used to assess effectivity of immunotherapy (52). Cattaneo et al. showed for example that effector function of autologous tumor-specific T cells could be assessed using peripheral blood-derived T cells and tumor organoids at a personalized level (53). Furthermore, efforts have been made to establish immune-engineered organoid models to study B-cell responses and antibody production (54, 55). Application of such combined organoid models to study (patient-specific) epithelial-inflammatory cell interactions in SSc-ILD, may provide an important step forward in our understanding of this devastating disease.

## Genetics

Accelerated lung aging and senescence of AEC and fibroblasts appear central phenotypes that promote lung fibrosis (8). Polymorphisms in genes involved in the maintenance of telomere length (mutations in TERT, TERC, PARN and RTEL1) are associated with an increased risk of IPF (56). Shortened telomeres, oxidative injury, endoplasmic reticulum (ER) stress, and mitochondrial dysfunction lead to decreased AEC proliferation and secretion of profibrotic mediators (8). In addition, genetic variants in the genes encoding e.g., surfactant protein C (SFPTC) (57, 58) and in the promotor region of mucin 5B (MUC5B), increase the risk of IPF development (59–61), while MUC5B has shown not to contribute to SSc-ILD (62). Whereas genetic susceptibility may be an important driver in IPF, for SSc, this is less clear. Several genetic factors may predispose to development of SSc, of which genetic variants in immune-related genes, including HLA and interferon-related genes, have been found (37). Genetic associations that predispose for development of SSc-ILD specifically, have been found in interferon regulatory factor 5 (IRF5), signal transducer and activator of transcription 4 (STAT4), DNAX accessory molecule 1 (CD226) and interleukin-1 receptor-associated kinase-1 (IRAK1) (63). Genetic variants in monocyte chemoattractant protein (MCP-1) and CTLA-4 were also associated with SSc-ILD (64). Especially, polymorphisms in CTLA-4, which acts as a checkpoint inhibitor, may be of relevance in SSc-ILD patients at risk for developing lung cancer.

Even though adult human cells can be isolated from lungs to study lung epithelial (progenitor) function, (altered) repair and regeneration, in SSc-ILD these are often derived from end-stage disease patients undergoing lung transplantation. To overcome this issue, human induced pluripotent stem cells (hiPSC)-derived lung epithelial cells may provide a personalized platform for assessing disease risk and potential treatment for SSc-ILD. One of the advantages of hiPSC-derived AEC2 is that they can be derived from individual patients from relatively easily accessible sources (urine, blood), enabling a personalized disease and medicine approach. Importantly, hiPSC-AEC2 can be genetically engineered by introducing or correcting mutations using CRISPR-based technology, which may be relevant to study genetic factors contributing to fibrosis in IPF and SSc-ILD (65). These hiPSC-derived AEC2 can be applied in lung organoids which can model pathogenetic processes occurring in ILD (66). Similar to hiPSC, lung organoids can also be derived from embryonic stem cells (ESC). A recent study used this approach with embryonic stem cells in which mutations were introduced in genes associated with Hermansky-Pudlak syndrome, that strongly predisposes to lung fibrosis, and thus demonstrated an essential role for IL-11 in the fibrotic process (67). Whereas generation of hiPSC- or ESC-derived AEC2 can be performed successfully, derivation of long-term primary AEC2 cultures from human lung tissue still is challenging. Recently, Shiraisi et al. successfully cultured AEC2 from human lungs using organoid expansion, which they were able to passage (68). More importantly, they transduced a pulmonary fibrosis-associated mutant SpC (SFPTC^Δexon4^) protein into AEC2, resulting in the development of similar AEC2 features also observed in IPF patients (68). Combinations of hiPSC-derived AEC2 and lung organoid models may therefore offer a personalized platform to study the contribution of genetic predisposition and environmental cues to fibrosis development in SSc.

## Biomarkers and Treatment

Markers of alveolar epithelial injury, including Krebs von den Lungen-6 (KL-6) and surfactant protein-D (SP-D) (27), are increased in serum and may serve as biomarkers in identifying and monitoring SSc-ILD (69, 70). Importantly, exhaled nitric oxide (Fe_NO_) has been proposed as a marker of (alveolar) inflammation in SSc-ILD. Fe_NO_ levels were shown to be increased in SSc compared to IPF patients, although it did not discriminate between SSc-patients with or without ILD (71, 72). In addition to the diagnostic potency of the circulating biomarkers and Fe_NO_, microvascular abnormalities are detectable before clinical presentation of fibrosis (28, 73), and may serve as a prognostic marker to monitor disease progression in subgroups of SSc-ILD patients. Furthermore, levels of smooth muscle cells and myofibroblasts are increased in SSc-ILD lung tissue compared to healthy controls, and may also contribute to fibrosis. Finally, pro-fibrotic mediators, including TGF-β, PDGF, CTGF and thrombin, are increased in bronchoalveolar lavage fluid (BALF) or cells derived from BALF (29) and may contribute to altered cellular composition and phenotype in SSc-ILD. Several other potential serum biomarkers are under current investigation, among which serum chemokine [C-C motif] ligand 2 and 18 (74, 75), matrix metalloproteinase-7 and 12 (76, 77) and chemokine [C-X-C motif] ligand 4 (78, 79). Because a complete overview of current serum biomarkers is beyond the scope of this review, we refer the interested reader to a recently published comprehensive review summarizing the clinically used biomarkers, biomarkers under investigation and their link to mechanistic pathways (80). Interestingly, lung organoid models may contribute to identification of novel biomarkers, as was shown by the identification of the essential profibrotic role of IL-11, upon introduction of genes associated with Hermansky-Pudlak syndrome (67).

In addition to providing mechanistic insight into SSc-ILD pathogenesis, and biomarker identification, preclinical lung organoid models may also be applied to identify new treatment targets and for (personalized) drug screening platforms. So far, treatment of SSc-.ILD is targeted at alveolitis and includes non-selective immunomodulatory drugs like cyclophosphamide, azathioprine and mycophenolate (81, 82), all of which target (T- and/or) B lymphocyte proliferation or trigger cell death (83), as B-lymphocytes have a pivotal role in the pro-inflammatory and profibrotic pathway in SSc-ILD. Standard therapy combined with rituximab, also targeting CD20 on B cells, was shown to be beneficial, with a lower decline of FVC (84), whereas moderate effects were seen on FVC levels and HRCT fibrosis scores (35). The observation that anti-inflammatory and immune suppressive treatment with e.g., prednisone and azathioprine (85), or administration of interferon gamma (86), are clinically not effective, suggests that (late/end-stage) IPF does not result from a primary immunopathogenic process. Recently approved antifibrotic drugs that target selected profibrotic mediators has improved the treatment of IPF (87). The most widely studied drugs in this category are pirfenidone and nintedanib, which were shown to be safe and effective in IPF treatment. Pirfenidone acts through anti-inflammatory and antifibrotic effects, including down-regulation of TGF-β and TNF-α (88), whereas. Nintedanib is a tyrosine kinase inhibitor that inhibits activation of e.g., VEGF, fibroblast growth factor (FGF), and PDGF pathways (89). As nintedanib was recently approved for IPF treatment (90), its effectivity was also examined in SSc-ILD patients (91). Nintedanib demonstrated a clear effect on the annual rate of decline in FVC in SSc-ILD patients (91, 92), suggesting that targeting common pro-fibrotic pathways of lung fibrosis may be of therapeutic relevance (28, 93). Therapeutic targets (under investigation) for IPF may be therefore also be considered for treatment of SSc-ILD.

Regarding treatments under evaluation for IPF, a potential therapeutic target is autotaxin, an enzyme responsible for lysophosphatidic acid (LPA) production (94), both of which are increased in IPF, suggesting that the autotaxin-LPA pathway has a pathogenic role in this disorder (95, 96). Results from a phase-2 trial with GLPG1690, targeting autotaxin, were encouraging, especially regarding decline in FVC (97). Clinical assessment of GLPG1690 as treatment for IPF patients is currently ongoing in a phase-3 trial. A phase 2 trial of pamrevlumab, targeting CTGF (16, 17), also showed a decreased rate in decline of forced vital capacity (FVC) as compared with placebo (98). However, the results of this phase-2 trial must be treated with caution, pending an adequately powered phase 3 study. Furthermore, pentraxin-2, which inhibits differentiation of monocytes toward profibrotic fibrocytes and inhibits TGF-β production (41, 44, 99), may be targeted to stabilize or restore lung function. Results from a phase-2 trial with pentraxin-2 showed a slower decline of FVC compared to placebo (99).

Other treatments that have been evaluated in SSc-ILD include abatacept, tocilizumab, and autologous stem-cell transplantation, and are reviewed elsewhere (6, 7, 84, 91). In daily practice, however, there is no consensus about the right timing of initiating anti-inflammatory and/or antifibrotic treatment and exact patient selection. Furthermore, up to date there are no accepted prognostic models available to identify SSc patients at high risk for the development of lung fibrosis (100). Lung organoid models may therefore be applied to identify new treatment targets and for (personalized) drug screening platforms. Importantly, these models may be used to examine the effect of combined therapies targeting pro-fibrotic and pro-inflammatory pathways simultaneously. Patient-derived lung cancer organoids, for example, were able to predict patient-specific drug responses and drug resistance (101). Such patient-specific models in the future may be used to test drug combinations, drug toxicity and identification of novel drug targets. Furthermore, new pre-clinical models may aid in discriminating SSc patients at high risk for development of ILD.

## Future Directions

Current concepts state that impaired or otherwise dysregulated repair of lung epithelial injury is an important driver of (progression of) fibrosis in SSc-ILD, but this is likely not unique to SSc-ILD. Delineating the triggers and/or the dysregulation of repair may reveal features that distinguish SSc-ILD from other ILD. Furthermore, exhaustion and dysfunction of progenitor cells may contribute to the failure in resolving damage. Pharmacological activation of lung progenitor cells may thus be a promising approach for treatment of especially early stage SSc-ILD.

To address these questions, a human lung model that recapitulates essential features of SSc-ILD is required to help understanding mechanisms underlying SSc-ILD and develop new treatments. Availability of such a model, that is also suitable for e.g., screening small-molecule libraries, would be a major step forward. In addition, such a model may provide novel tools for personalized medicine to identify those that may benefit from an anti-inflammatory or an antifibrotic treatment or combined treatment strategies. The need for this is further illustrated by the recent introduction of the antifibrotic drug nintenadib in the treatment of SSc-ILD patients, based on clinical trials showing clear efficacy with a similar slowdown in the rate of lung function decline as found in IPF patients treated with antifibrotic agents (91). SSc-ILD patients with progressive fibrosis may therefore be selected for treatment with these drugs.

**FIGURE 2:**
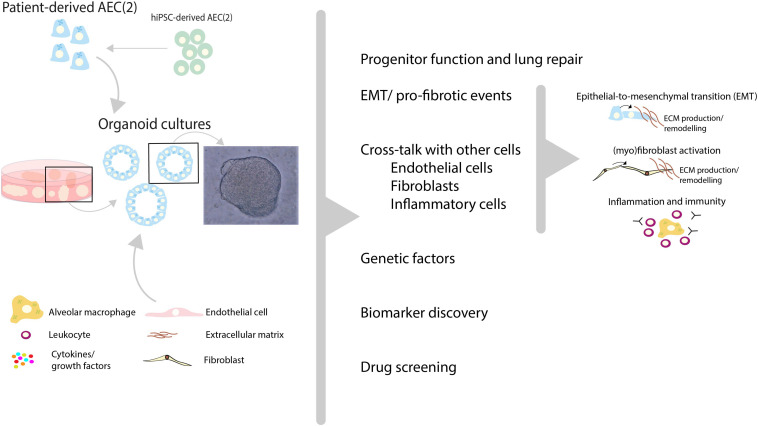
Recent advances in human lung organoid models may help to unravel pathogenic mechanisms and new treatment targets of SSc-ILD. Patient-derived pulmonary cells, either isolated from lung tissue or derived from human induced pluripotent stem cells (hiPSC), can be expanded *in vitro* using 3D organoid culture models. In these models, a variety of (disease-)relevant characteristics including progenitor cell function, profibrotic features, interactions with other cells (endothelial cells, fibroblasts etc.) and inflammatory cell recruitment can be studied. In addition, in such models the contribution of genetic factors to SSc-ILD can be studied using either patient-derived cells with specific genetic features, or by genetic editing of primary AEC (2) or hiPSC-derived AEC. These 3D lung organoid cultures can be applied to study various aspects of SSc-ILD pathogenic mechanisms and may therefore provide patient-relevant models which may contribute to biomarker and target discovery and aid in personalized drug screening for SSc-ILD.

The concept of using organoid cultures for selecting individual patients for the optimal treatment is illustrated by the recent publication by Berkers and co-workers (102), in which rectal organoids grown from stem cells provide a tool for personalized medicine in patients suffering from cystic fibrosis. Organoids in this study showed a high correlation between *in vitro* and *in vivo* effects of drugs and demonstrated good to excellent predictive value for preclinical identification of response to treatment. Studies such as those by Strikoudis et al., showed that organoids can also be used to study fibrotic lung disease, indicating that organoid technology may help to understand underlying mechanisms of SSc-ILD (Figure 2) (67).

Important features of fibrotic lung diseases include matrix abnormalities, leading to increased tissue stiffness and consequent altered mechanical forces to which pulmonary cells are exposed, resulting in e.g., increased TGF-β signaling and fibroblast activation (46, 103). These alterations can be mimicked with state-of-the art organs-on-chips (including lung-chips) platforms, which provide us with unprecedented combinations of important features in the lung, including matrix stiffness, mechanical force (breathing pattern), air exposure and co-culture with various relevant cell types (5, 104). Combining organoid and organ-on-chip technology may lead to better patient-relevant culture models, which can be used for drug screening purposes and research into the pathogenesis.

In addition to these advances in *in vitro* modeling, there are also important developments in analysis of cells in lung tissue that can also be applied in the analysis of the above-mentioned cellular models. Patient-derived cells can for instance be analyzed using single-cell-based RNA sequencing, which has already resulted in the identification of previously undescribed cell populations in SSc-ILD (105). Furthermore, patient-derived cells can now be analyzed at the protein level using (imaging) mass cytometry, enabling multidimensional unbiased analysis of more than 40 markers simultaneously (106) and an unprecedented potential for discovery of molecular culprits of disease. Mass cytometry analyses already revealed distinct immune cell signatures in patients with SSc (107).

In conclusion, the mechanisms underlying pulmonary fibrosis in SSc-ILD are not completely unraveled, and knowledge on fibrotic processes have been acquired mostly from studies in idiopathic pulmonary fibrosis (IPF). Recent advances in human preclinical models and biomedical technology may be used to unravel pathogenic mechanisms and new treatment targets of SSc-ILD (summarized in Figure 1). Developments in *in vitro* modeling using patient-derived organoid-based culture models and lung-chip platforms, as well as single cell analysis using e.g., single-cell RNA sequencing and (imaging) mass cytometry will be important to further broaden our insight in SSc-ILD pathogenesis and develop better and personalized treatments.

## Author Contributions

PK and EM drafted the manuscript. PK prepared the figures. PK, EM, PH, MN, and MG edited and revised the manuscript, and approved the final version of the manuscript. All authors contributed to the article and approved the submitted version.

## Conflict of Interest

The authors declare that the research was conducted in the absence of any commercial or financial relationships that could be construed as a potential conflict of interest.
